# Evaluation of the correlation between multiple organ calcification on CT and disease severity in patients with TAFRO syndrome

**DOI:** 10.1007/s11604-023-01394-2

**Published:** 2023-02-02

**Authors:** Rui Kano, Takao Igarashi, Ryo Kikuchi, Hiroya Ojiri, Atsushi Katsube, Shingo Yano

**Affiliations:** 1 Department of Radiology, The Jikei University School of Medicine, 3-25-8, Nishi-Shimbashi, Minato-Ku, Tokyo, 105-8461 Japan; 2 Department of Pathology, The Jikei University School of Medicine, 3-25-8, Nishi-Shimbashi, Minato-Ku, Tokyo, 105-8461 Japan; 3 Division of Clinical Oncology/Hematology, Department of Internal Medicine, The Jikei University School of Medicine, 3-25-8, Nishi-Shimbashi, Minato-Ku, Tokyo, 105-8461 Japan

**Keywords:** Adrenal glands, Myocardium, Calcification, X-ray computed tomography

## Abstract

**Purpose:**

The purpose of this study was to investigate the incidence of multiple organ calcification and the correlation between multiple organ calcification and clinical severity in patients with thrombocytopenia, anasarca, fever, reticulin fibrosis, renal dysfunction, and organomegaly (TAFRO) syndrome.

**Methods:**

We retrospectively identified 13 patients with TAFRO syndrome who were treated at our hospital between February 2019 and March 2021. Computed tomography (CT) images of TAFRO patients, which were acquired at admission and one month after admission, were evaluated. Additionally, clinical and laboratory data related to organ calcification and severity classification of TAFRO syndrome were investigated. The correlation between the presence of organ calcification on CT and TAFRO syndrome-severity classification was evaluated.

**Results:**

One month after admission, calcification of the myocardium, adrenal glands, gallbladder wall, pancreas, kidney, skeletal muscle, and skin were observed in 38%, 46%, 15%, 15%, 15%, 23%, and 15% of the thirteen patients, respectively. The occurrence rate of calcifications in the myocardium, adrenal glands, and skeletal muscle was significantly higher in patients with a grade 4 or higher clinical severity than in those with a level up to grade 3 (*p* = 0.001, *p* = 0.005, and *p* = 0.035, respectively).

**Conclusions:**

Our results suggest that the higher the clinical severity in patients with TAFRO syndrome, the higher is the frequency of calcification in the myocardium, adrenal glands, and skeletal muscle; therefore, the assessment of these organ calcifications on CT images may be useful in predicting the severity of TAFRO syndrome.

## Introduction

Thrombocytopenia, anasarca, fever, reticulin fibrosis, renal dysfunction, and organomegaly (TAFRO) syndrome is a new disease variant reported by Takai et al. in 2010 [1]. The pathophysiology of TAFRO syndrome is a systemic inflammatory response, and pathological similarities with multicentric Castleman disease (MCD) have been reported [1–4]; however, the definite pathophysiological mechanism causing TAFRO syndrome remains unclear, and many unknown aspects exist in the clinical and diagnostic imaging of the disease.

A few characteristic computed tomography (CT) imaging findings that are suggestive of TAFRO syndrome have been reported in recent years [5–9]. Non-mass-forming infiltrative lesions on the anterior mediastinum, para-aortic oedema, and adrenal haemorrhage or adrenal enlargement have been reported as characteristic imaging findings suggestive of TAFRO syndrome, which are rarely observed in other diseases [5–9]. Moreover, these imaging findings are useful for the early diagnosis and subsequent appropriate therapeutic management of TAFRO syndrome [5, 6].

Recently, a new imaging finding involving myocardial and skeletal-muscle calcification was reported for a patient with TAFRO syndrome [10]. In clinical practice, myocardial calcification is occasionally seen in patients with old myocardial infarction. Except for the patients having coronary artery disease, myocardial calcification is relatively rare, and there are only a few case reports of myocardial calcification secondary occurring after septic shock other than in TAFRO syndrome [11–13]; therefore, calcification may be the imaging finding to be evaluated when assessing disease status in patients with TAFRO syndrome. In addition, TAFRO syndrome may cause calcification in multiple organs besides myocardium and skeletal muscle; however, there are no reports yet regarding this issue. The relationship between calcification and the clinical severity of TAFRO syndrome has also not been clarified.

The aim of our study was to investigate the incidence of organ calcification and the correlation between the presence of organ calcification and clinical severity in patients with TAFRO syndrome.

## Materials and methods

This was a retrospective, single-centre, observational study. The relevant institutional review boards approved this study, and the requirement for informed consent was waived.

### Patients

Our study included 13 Japanese patients, who were admitted at our hospital between February 2019 and March 2021, and met the 2019 updated diagnostic criteria for TAFRO syndrome [14].

### CT image acquisition

All CT examinations were performed using a 128-section multi-detector CT system (SOMATOM Definition AS or SOMATOM Drive, Siemens Healthineers, Erlangen, Germany) at the end of inspiration, with or without contrast medium administration. Axial images were acquired at 5 mm thickness using a 120 kV peak (kVp) and 120–460 milliarcsecond (mAs). Chest-to-pelvis CT image acquisitions were performed at least twice for all patients on admission and one month after admission. Mean day from symptom onset to the first CT examination was 31.2 days, with a range of 1–110 days. The first CT examination was performed before the initiation of treatment and dialysis, and the second after the initiation of treatment and dialysis.

### Evaluation of diagnostic criteria and severity classification for TAFRO syndrome

A radiologist with 5 years of experience retrospectively acquired data on patients with TAFRO syndrome from our institutional database. All included patient data had been assessed by haematological oncologists at our institution at the time of patients’ admission to the hospital using Masaki’s criteria, which were updated in 2019, as the diagnostic criteria for TAFRO syndrome in this study. Masaki’s criteria require all three major categories: (1) anasarca, including pleural effusion, ascites, and general oedema; (2) thrombocytopenia; platelet count < 100,000/µL, without myelosuppressive treatment; (3) systemic inflammation, defined as fever of unknown aetiology above 37.5 °C and/or serum C-reactive protein concentration (CRP) > 2 mg/dL and at least two of four minor categories: (1) Castleman disease-like features in lymph-node biopsy; (2) reticulin myelofibrosis and/or an increased number of megakaryocytes in the bone marrow; (3) mild organomegaly, including hepatomegaly, splenomegaly, and lymphadenopathy, and (4) progressive renal insufficiency. The exclusion criteria were (1) malignancies, including lymphoma, myeloma, and mesothelioma; (2) autoimmune disorders, including systemic lupus erythematosus (SLE), Sjogren’s syndrome, and ANCA-associated vasculitis; (3) infectious disorders, including acid-fast bacterial infection, rickettsia disease, Lyme disease, severe fever with thrombocytopenia syndrome (SFTS); (4) polyneuropathy, organomegaly, endocrinopathy, monoclonal gammopathy, and skin changes (POEMS) syndrome; (5) hepatic cirrhosis, and (6) thrombotic thrombocytopenic purpura (TTP)/haemolytic uremic syndrome (HUS) [14].

The severity classification for TAFRO syndrome is determined by calculating the summation of the number of points based on the symptoms present; the points were allocated as: (1) anasarca: one point each for pleural effusion on imaging, ascites on imaging, and pitting oedema on physical examination; (2) thrombocytopenia: one point for a platelet count below 100,000/µL, two points for a platelet count below 50,000/µL, and three points for a platelet count below 10,000/µL, up to three points; (3) fever and/or inflammation: one point for a fever between 37.5 °C and 38 °C or for CRP concentration between 2 and 10 mg/dL, two points for a fever between 38 °C and 39 °C or for CRP concentration between 10 and 20 mg/dL, three points for fever exceeding 39 °C or for CRP exceeding 20 mg/dL, up to three points; (4) renal insufficiency: one point for a glomerular filtration rate (GFR) < 60 mL∙min^−1^/1.73 m^2^, two points for GFR < 30 mL∙min^−1^/1.73 m^2^, three points for GFR < 15 mL∙min^−1^/1.73 m^2^ or in need of haemodialysis, up to three points. The relationship between total score and disease severity was determined as follows: 0–4 points: mild (grade 1); 5–6 points: moderate (grade 2); 7–8 points: slightly severe (grade 3); 9–10 points: severe (grade 4); 11–12 points: very severe (grade 5). Although several methods for assessment exist for anasarca, we evaluated it using chest and abdominal CT images on admission [14].

Patients were divided into two groups based on the severity classification applicable to them: one group included patients with mild (grade 1), moderate (grade 2), and slightly severe (grade 3) TAFRO syndrome, and the other group included those with severe (grade 4) and very severe (grade 5) TAFRO syndrome.

### Clinical and laboratory data

In this study, in addition to the diagnostic criteria and severity classification for TAFRO syndrome, we collected the following clinical and laboratory data on the first day of hospitalisation; these were considered indicators of the occurrence of calcification: plasma calcium (normal range, 8.8–10.1 mg/dL), plasma inorganic phosphorus (normal range, 2.7–4.6 mg/dL), plasma alkaline phosphatase (plasma ALP; normal range, 106–322 U/L), creatinine (Cr; normal range, 0.65–1.07 mg/dL), and haemoglobin (Hb; normal range, 13.7–16.8 g/dL). Intact parathyroid hormone (iPTH; normal range, 10–65 pg/mL) was recorded if it had been measured.

### Clinical outcome

Based on the clinical outcome of 13 patients, they were divided into two groups, remission or deceased, depending on whether they recovered and were discharged from our institution after treatment, or died owing to lack of response to treatment, respectively.

### Evaluation of CT imaging findings

Axial CT imaging findings were retrospectively evaluated by two radiologists with 5 years and 20 years of experience, each using a picture-archiving and communication system. Calcification was determined positive when high-attenuation areas were detected and a CT value above 250 Hounsfield units was observed. Differences in interpretations were resolved via consensus reading.

The presence of organ calcification on CT imaging was evaluated on admission and one month after admission. In the case of paired organs such as the adrenal glands or kidneys, calcification was considered present even if it was observed only on one side.

### Pathological examination

A pathological examination was performed on four lymph-node biopsies after admission and three autopsy patients diagnosed by experts in the pathology department of our institution, and they were blinded to the CT findings. Tissue sections of 5 μm thickness were prepared from formalin-fixed and paraffin-embedded tissue samples.

Each tissue section was processed for haematoxylin and eosin (HE) staining. Direct fast scarlet staining and polarised light microscopy were used to detect amyloid deposition for specimens in which calcification was observed.

### Statistical analysis

Fisher’s exact test was used to analyse the binary variables. All statistical analyses were performed using SPSS version 25 (IBM Corporation). Statistical significance was set at *p* < 0.05.

## Results

**Fig. 1:**
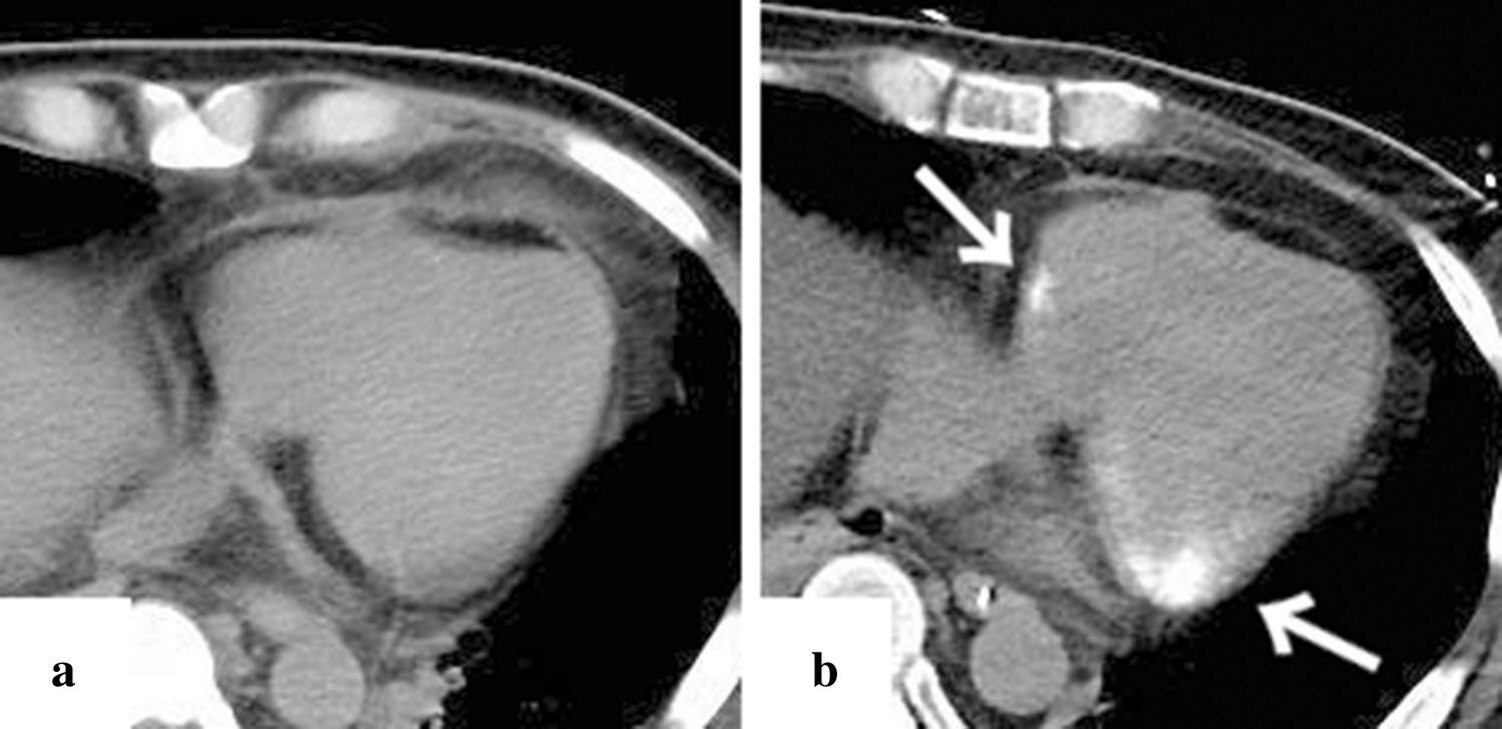
Representative case of myocardial calcification in a 51-year-old patient (case no. 13). Computed tomography (CT) imaging on day 1 since the onset of symptoms (**a**) and after one month of the admission (**b**). Calcification occurred in the myocardium (**b**, white arrow), and there was no calcification in the coronary arteries

**Fig. 2:**
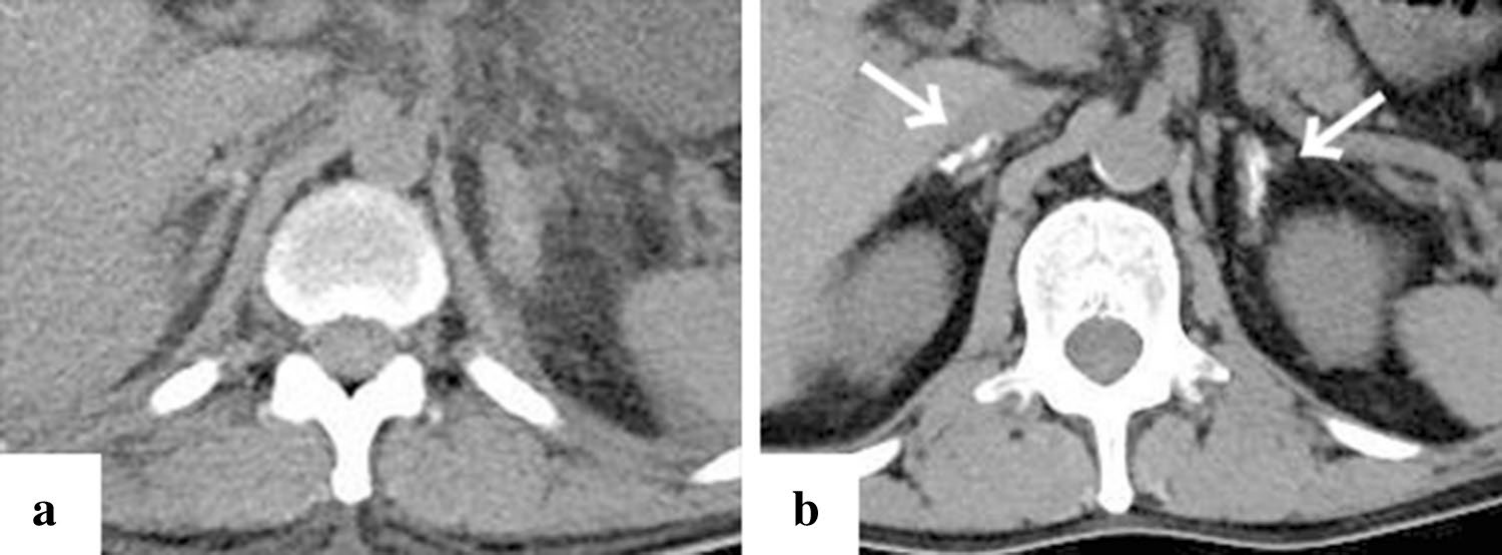
Representative case of adrenal calcification in a 50-year-old patient (case no. 2). Computed tomography (CT) imaging on day 41 since the onset of symptoms (**a**) and after one month of the admission (**b**). CT imaging on admission indicated adrenal enlargement (**a**), and CT one month later adrenal calcification (**b**, white arrow)

**Fig. 3:**
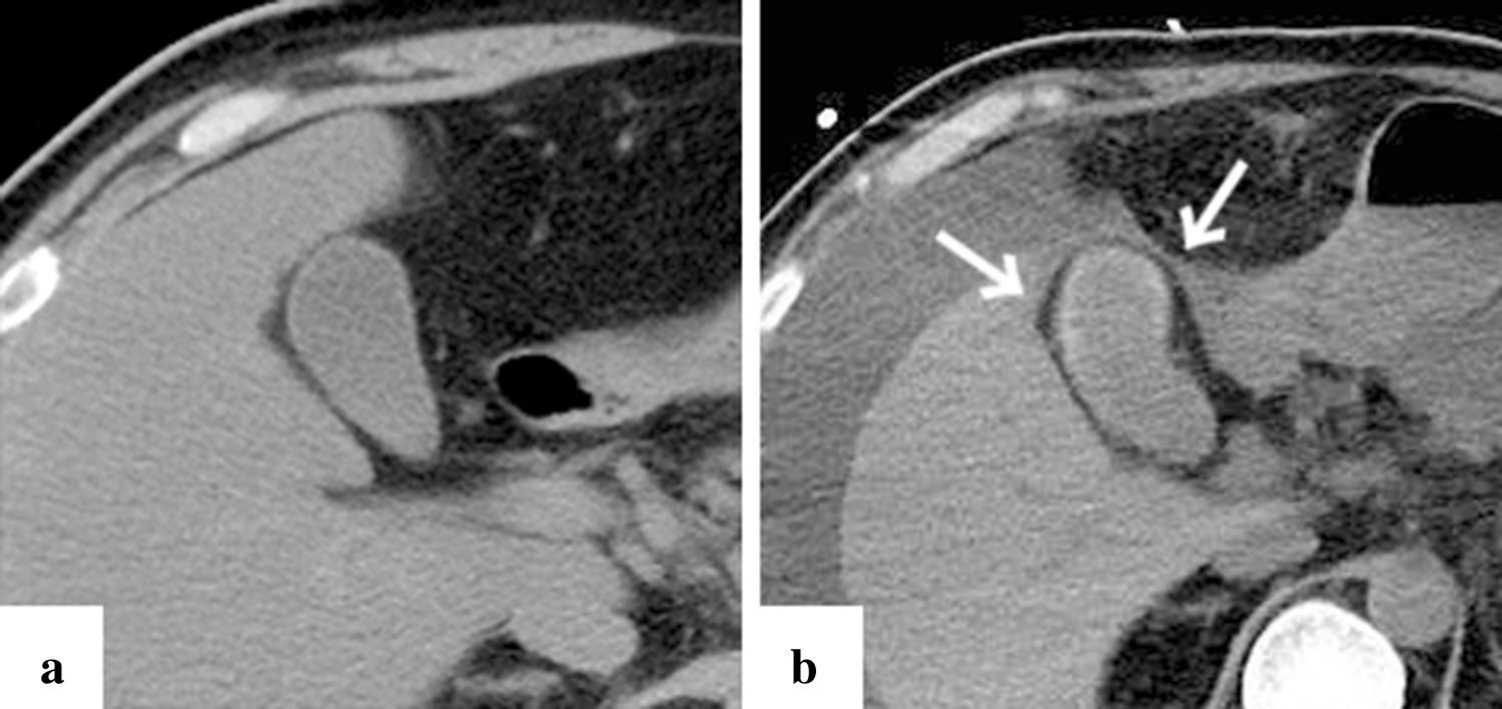
Representative case of gallbladder calcification in a 51-year-old patient (case no. 13). Computed tomography (CT) imaging on day 1 since the onset of symptoms (**a)** and after one month of the admission (**b**). One month after admission, thickening of the gallbladder wall was not observed and calcification of the wall occurred mainly at the base of the gallbladder (**b**, white arrow)

**Fig. 4:**
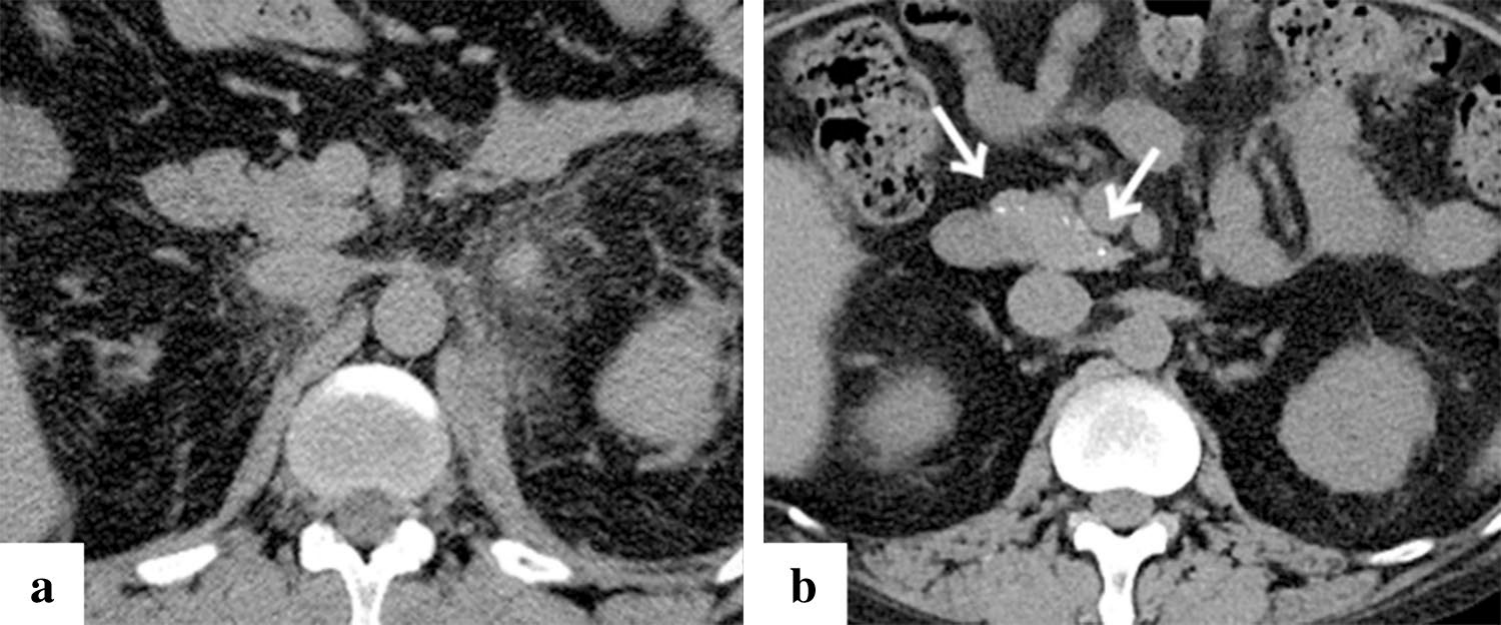
Representative case of pancreatic calcification in a 43-year-old patient (case no. 8). Computed tomography (CT) imaging on day 11 since the onset of symptoms (**a)** and after one month of the admission (**b**). CT imaging on admission showed no evidence of acute inflammation in the pancreas (**a**), and CT imaging one month after admission revealed calcification of the pancreas (**b**, white arrow). In addition, CT imaging on admission showed bilateral retroperitoneal oedema (**a**)

**Fig. 5:**
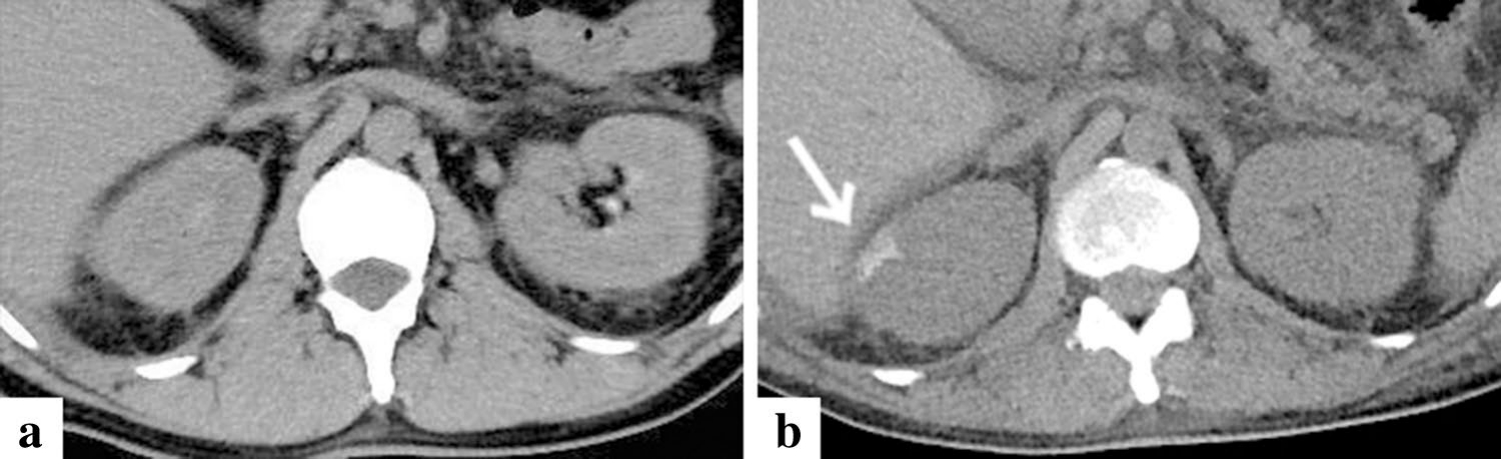
Representative case of renal calcification in a 50-year-old patient (case no. 2). Computed tomography (CT) imaging on day 41 since the onset of symptoms (**a)** and after one month of the admission (**b**). CT imaging on admission showed fatty tissue turbidity around the bilateral kidneys due to acute renal injury from TAFRO syndrome (**a**). One month after admission, CT imaging showed improvement in the finding of fatty tissue turbidity around the bilateral kidneys; however, calcification appeared in the kidney (**b**, white arrow)

**Fig. 6:**
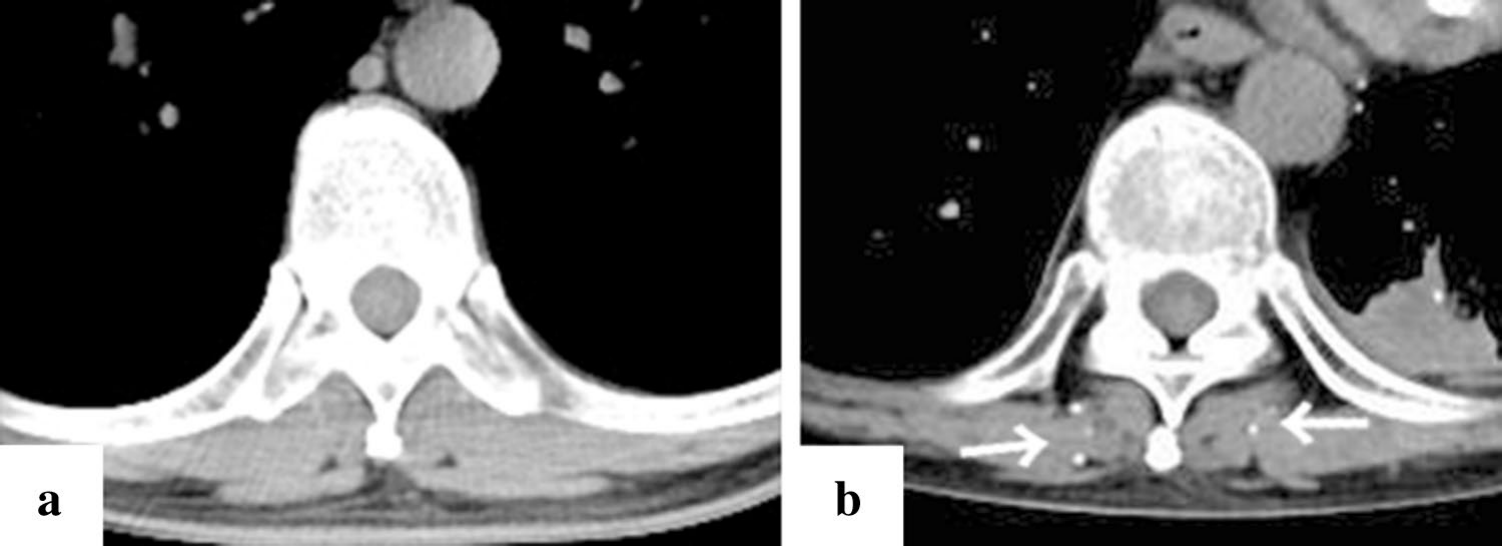
Representative case of skeletal muscle calcification in a 41-year-old patient (case no. 12). Computed tomography (CT) imaging on day 3 since the onset of symptoms (**a**) and after one month of the admission (**b**). CT imaging on admission showed no abnormalities in the skeletal muscle (**a**), and CT imaging one month after admission showed calcification in the skeletal muscle (**b**, white arrow)

**Fig. 7:**
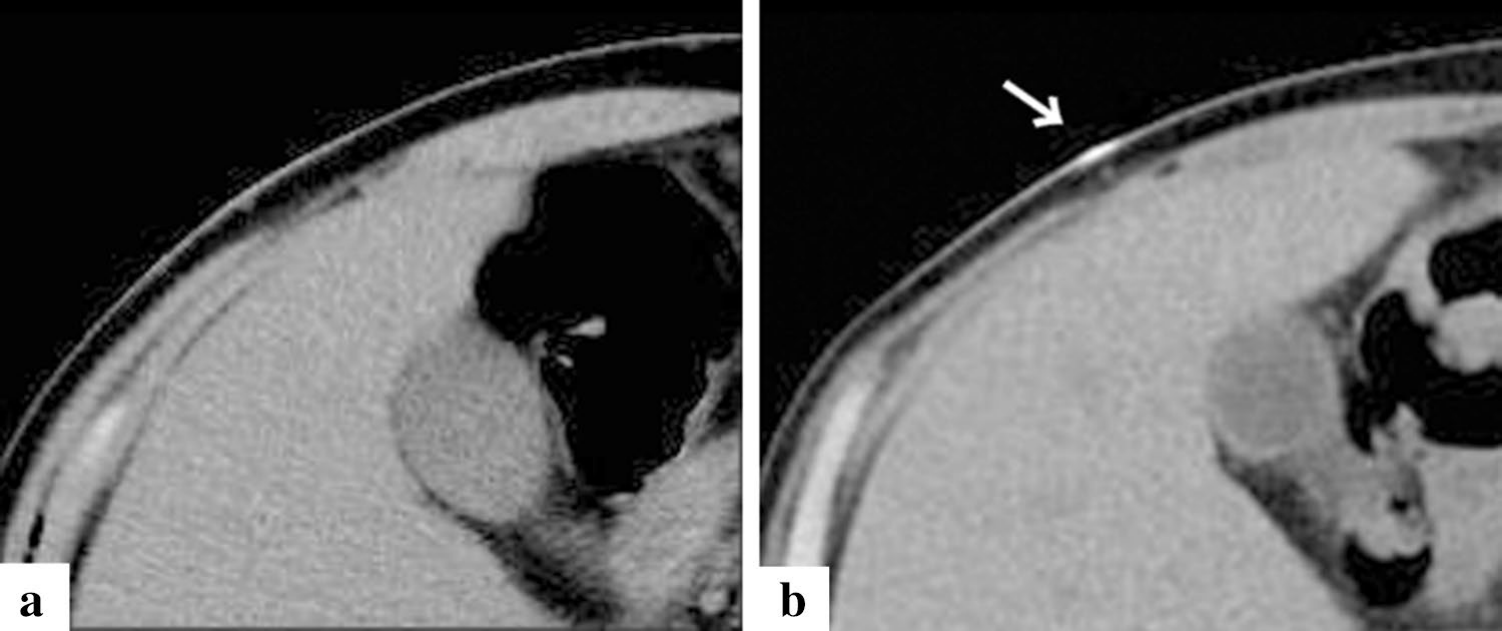
Representative case of dermal calcification in a 50-year-old patient (case no. 2). Computed tomography (CT) imaging on day 41 since the onset of symptoms (**a**) and after one month of the admission (**b**). CT imaging on admission showed no abnormalities in the skin (**a**), and CT imaging one month after admission showed calcification in the skin (**b**, white arrow)

A total of 13 patients (10 men and 3 women), with an average age of 55.2 years (range 36–84 years) were included in this study. The results are summarised in Table 1. At the time of hospitalisation, the number of patients with disease severity grades 1, 2, 3, 4, and 5 was 0 (0%), 6 (46%), 2 (15%), 3 (23%), and 2 (15%), respectively. None of the patients exhibited any organ calcification at this time. One month after admission, CT imaging revealed calcification in 38% (5/13), 46% (6/13), 15% (2/13), 15% (2/13), 15% (2/13), 23% (3/13), and 15% (2/13) of patients in their myocardium, adrenal glands, gallbladder wall, pancreas, kidney, skeletal muscle, and skin, respectively (Figs. 1, 2, 3, 4, 5, 6, and 7). In all patients, the calcifications deposited in the adrenal gland and kidney were bilateral. In addition, all cases with adrenal calcification one month after admission were identified via CT findings suggesting haemorrhage or enlargement of the bilateral adrenal glands on admission (Fig. 2). All patients with calcification in organs other than the adrenal glands had a disease severity of grade 4 or higher. One of the two patients with grade 3 diseases exhibited calcification only in the adrenal glands. All patients with disease severity of grade 4 or higher had hyperphosphatemia, and all but one had elevated serum iPTH. Hypercalcemia was present in only one patient with disease severity of grade 4 or higher. There was no correlation between the severity of TAFRO syndrome or organ calcification and the clinical outcome. The patient with case no. 12 had a positive blood culture and met the diagnostic criteria for septic shock, from which he recovered with antibiotic therapy in addition to the treatment for TAFRO syndrome. Cerebral haemorrhage was the cause of death in cases no. 3 and no. 5, and multiple organ failure in cases no. 8 and no. 13. One (case no. 12) of the patients with myocardial calcification suffered a cardiac arrest during rehabilitation and survived after cardiopulmonary resuscitation. The patient had repeated attacks of loss of consciousness, which turned out to be caused by severe atrioventricular block. Hence, a permanent pacemaker was implanted.

**Table 1:** CT imaging and laboratory factors of 13 patients with TAFRO syndrome

Case no	1	2	3	4	5	6	7	8	9	10	11	12	13	Detection rate (*n*)
Age (year)/sex	55/M	50/M	66/M	45/M	67/M	36/F	60/M	43/M	64/M	55/F	84/F	41/M	51/M	
Disease severity classification														
Anasarca	2	3	3	3	3	3	3	3	3	3	3	3	3	
Thrombocytopenia	1	2	2	2	1	1	1	2	1	2	2	1	2	
Fever and/or inflammation	1	3	1	3	1	1	1	2	1	1	1	3	3	
Renal insufficiency	1	3	2	3	0	0	1	3	0	1	0	3	2	
Total points	5	11	8	11	5	5	6	10	5	7	6	10	10	
Disease severity (Grade)	2	5	3	5	2	2	2	4	2	3	2	4	4	
Calcification														
Myocardium	−	+	−	+	−	−	−	+	−	−	−	+	+	38% (5/13)
Adrenal grand	−	+	+	+	−	−	−	+	−	−	−	+	+	46% (6/13)
Gall bladder	−	−	−	−	−	−	−	+	−	−	−	−	+	15% (2/13)
Pancreas	−	−	−	−	−	−	−	+	−	−	−	+	−	15% (2/13)
Kidney	−	+	−	−	−	−	−	−	−	−	−	−	+	15% (2/13)
Skeletal muscle	−	+	−	−	−	−	−	−	−	−	−	+	+	23% (3/13)
Skin	−	+	−	−	−	−	−	−	−	−	−	+	−	15% (2/13)
Laboratory factors														
Hypercalcemia [Ca > 10.2 mg/dL]	−	−	−	−	−	−	−	−	−	−	−	−	+	8% (1/13)
Hypocalcemia [Ca < 8.5 mg/dL]	−	−	−	−	−	−	−	−	−	−	−	−	−	
Hyperphosphatemia [IP > 4.7 mg/dL]	−	+	+	+	−	+	−	+	−	−	−	+	+	54% (7/13)
Hypophosphatemia [IP < 2.5 mg/dL]	−	−	−	−	−	−	−	−	−	−	−	−	−	
PTH [> 65 pg/mL]	N/A	−	N/A	+	N/A	N/A	N/A	+	N/A	N/A	N/A	+	+	80% (4/5)
ALP [> 323 U/L]	−	+	−	+	+	−	+	+	+	+	+	+	+	77% (10/13)
Cr [> 1.08 mg/dL]	+	+	+	+	−	−	+	+	−	+	−	+	+	62% (8/13)
Hb [< 13.6 g/dL]	+	+	+	+	+	+	+	+	+	+	+	+	+	100% (13/13)
Dialysis	−	+	+	+	−	−	−	+	−	−	−	+	+	46% (6/13)
Treatment	PSL + CyA + TOC	PSL + CyA	PSL + CyA	PSL + CyA	PSL + CY + Tac + RIT + IVIG	PSL	PSL	PSL + CyA + TOC	PSL	PSL	PSL	PSL + CyA	PSL + CyA + RIT	
Clinical course and outcome	Remission	Remission	Deceased	Remission	Deseased	Remission	Remission	Deseased	Remission	Remission	Remission	Remission	Deseased	

*M* male, *F* Female, *N/A* not applicable, *PTH* parathormone, *ALP* alkaline phosphatase, *Cr* creatinine, *Hb* haemoglobin, *PSL* prednisolone, *CyA* CyclosporineA, *TOC* tocilizumab, *CY* cyclophosphamide, *Tac* tacrolomus, *RIT* rituximab, *IVIG* intravenous immunoglobulin

The results of Fisher’s exact test for the presence of calcification detected one month after admission in the groups of grade 3 or less (mild, moderate, and slightly severe) and grade 4 or more (severe and very severe) are summarised in Table 2. The occurrence rates of calcification in the myocardium, adrenal glands, and skeletal muscle were significantly higher in patients with grade 4 or higher than in those with grade 3 or lower. Although not significant, all cases with calcifications in organs other than the adrenal glands were classified as grade 4 or higher.

**Table 2:** Calcification of CT imaging with TAFRO syndrome one month after admission

Calcification of CT imaging	Number of positive cases		*P* (Fisher’s exact test)
	Grade 1, 2, 3 (*n* = 8)	Grade 4, 5 (*n* = 5)	
Myocardium	0	5	*0.001*
Adrenal gland	1	5	*0.005*
Gall blader	0	2	0.128
Pancreas	0	2	0.128
Kidney	0	2	0.128
Skeletal muscle	0	3	*0.035*
Skin	0	2	0.128

Italic *P* values (*P*) mean statically significant

**Fig. 8:**
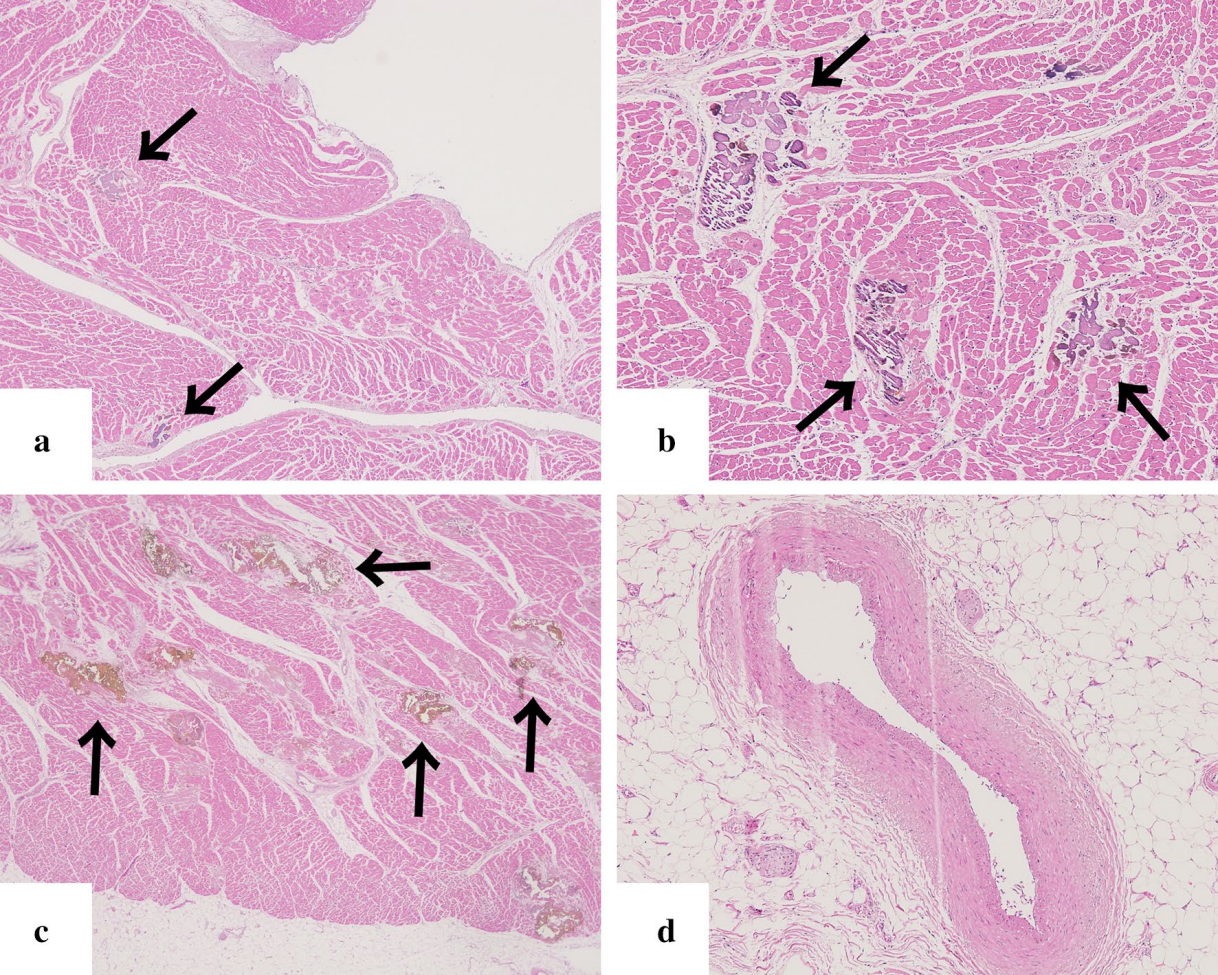
Left ventricular myocardium obtained in autopsy (case no. 13; HE stain). Calcification was sparsely distributed throughout the layers of myocardium, with no evidence of uneven distribution in the endocardial or epicardial side. (**a**: endocardial side**, b**: middle layer of myocardium**, c**: epicardial side). Calcification (**a**, **b**, **c**; black arrows) was distributed within myocardial fibre, and no necrosis of myocardium was observed. No calcification was observed in the coronary arteries (**d**: coronary artery)

Four patients (cases no. 3, no. 6, no. 7 and no. 13) had lymph-node biopsies performed after admission, and all the pathological examinations indicated lymph node with follicular plasma-cell proliferation. Autopsies were performed on three patients (cases no. 3, no. 8 and no. 13), two of whom had calcifications in the myocardium (case no. 8 and no. 13). Calcifications were detected in the myocardia of both atria and ventricles on pathological examination (Fig. 8) (case no. 13). Direct fast scarlet staining yielded a negative result, and birefringence was not observed using polarised light microscopy.

## Discussion

Calcifications were detected in all patients with grade 3 or higher (6 of 13 patients) severity on CT at one month after admission. The adrenal glands showed the highest occurrence of calcification, followed by the myocardium. Our results suggest that the higher the severity of classification in TAFRO syndrome, the higher is the frequency of calcification in the myocardium, adrenal glands, and skeletal muscle.

Calcification of a blood-vessel wall is frequently detected on CT examination for patients on dialysis or with atherosclerosis, whereas it is rarely detected in parenchymal organs, such as the adrenal glands, the myocardium, and/or the skeletal muscle. The mechanism of organ calcification can be divided into metastatic and dystrophic processes, which are caused by abnormalities in calcium or phosphate metabolism and by cellular damage or necrosis induced by infarction, trauma, inflammation, infection, and drugs, respectively [10, 15]. In addition, metastatic calcification occurs gradually over a period spanning months or even years, whereas dystrophic calcification occurs rapidly, sometimes in a matter of days to weeks [11, 12]. Calcification of the adrenal glands is often encountered after a haemorrhage, infarction, or tuberculosis infection [6, 16]. A recent retrospective cohort study reported that adrenal abnormalities in TAFRO syndrome are likely related to congestive infarction associated with venous or lymphatic congestion because para-aortic venous and/or lymphatic drainage deteriorates for unknown reasons, or a catecholamine released from the adrenal glands may promote vasoconstriction and platelet aggregation, resulting in vasospasm and haemorrhage of the fragile adrenal capillary vessels [6, 8]. Although the pathogenesis of adrenal abnormalities is beyond speculation, we believe that the aetiology of adrenal calcification following adrenal abnormalities is most likely dystrophic calcification in patients with TAFRO syndrome. All cases with adrenal calcification one month after admission were identified via CT findings, suggesting haemorrhage or enlargement of the bilateral adrenal glands on admission (Fig. 2). A patient exhibiting myocardial calcification in CT findings is rarely encountered in clinical practice. Several cases of dystrophic calcification of the myocardium secondary to septic shock have been reported [11–13], and myocardial calcification is associated with cardiac arrest [10, 12]. One of the patients in our study who developed myocardial calcification had a cardiac arrest. All CT findings of myocardial calcification in our study were similar to those CT images observed in septic shock described in case reports (Fig. 1) [11–13]. Diffuse myocardial damage is assumed to occur because of acute-onset hypotension, leading to dystrophic calcium deposition in impaired or necrotic cells [11, 13, 17]. Furthermore, the use of pressor therapy in patients with shock may induce dystrophic calcification within weeks because high catecholamine levels can lead to myocardial ischaemia and necrosis [11, 13]. In our study, all patients who developed myocardial calcification were critically severe or very severe and required renal replacement therapy and vasopressors for haemodynamic support during the acute phase of illness. One of the patients with myocardial calcification had a positive blood culture and met the diagnostic criteria for septic shock, whereas the other four patients had no infectious complications (case no. 12). Based on the above, hypotension associated with haemodynamic collapse due to TAFRO syndrome and the use of vasopressors could be the cause of dystrophic myocardial calcification. Most patients with myocardial and adrenal calcifications also had calcification in other parenchymal organs (Figs. 3, 4, 5, 6 and 7). Calcification of the pancreas and gallbladder is an occasional finding in chronic pancreatitis and porcelain gallbladder; however, it is very rare for simultaneous and rapid occurrence including kidney, skeletal muscle, and skin. Minomo suggested that metastatic calcification due to progressive renal dysfunction and hypocalcemia is a cause of calcification in cardiac and skeletal muscle with a background of TAFRO syndrome [10]. Although none of the patients in our study had hypocalcemia, all the patients with multi-organ calcification had progressive renal dysfunction and hyperphosphatemia; therefore, the mechanism of calcification in the gallbladder, pancreas, kidney, skeletal muscle, and skin was considered more likely to be metastatic than dystrophic. Given these facts, we infer that both metastatic and dystrophic calcification may have been involved in the process of producing calcification of the adrenal gland and myocardium. Considering the seriousness of complications such as cardiac arrest owing to arrhythmia and/or restrictive cardiomyopathy that may arise in patients with myocardial calcification [10, 12], we believe that when myocardial calcification is confirmed on CT imaging for patients with TAFRO syndrome, the assessment of cardiac function using electrocardiography and echocardiography is necessary to prevent such life-threatening complications.

Our study has some limitations. First, our study was retrospective and used consensus reading. Second, the number of patients was small because TAFRO syndrome is a rare disease. Third, the disease severity classification used in our study is original to the authors and may not apply to all patients with TAFRO syndrome. Fourth, although an evaluation of the relationship between disease severity and quantitative assessment of imaging is generally expected, a quantitative analysis of organ calcification on CT was not performed herein. Finally, specimens of myocardial calcification could be obtained from only three cases at autopsy. Consequently, the pathogenesis of this condition has not been elucidated.

In summary, we observed that patients with severe TAFRO syndrome may frequently develop calcification of parenchymal organs, particularly in myocardium, adrenal glands, and skeletal muscle. Our results suggest that the assessment of these organ calcifications on CT images may be useful in predicting the severity of TAFRO syndrome. In particular, early detection and follow-up of myocardial calcification on CT may assist in predicting the possibility of occurring arrhythmias and cardiac arrest in patients with severe TAFRO syndrome.

## Abbreviations

ALP: Alkaline phosphatase
ANCA: Anti-neutrophil cytoplasmic antibody
Cr: Creatinine
CRP: C-reactive protein concentration
CT: Computed tomography
GFR: Glomerular filtration rate
Hb: Haemoglobin
HE: Haematoxylin and eosin
HUS: Haemolytic uremic syndrome
kVp: Kilovoltage peak
mAs: Milliampere-second
MCD: Multicentric Castleman disease
POEMS: Polyneuropathy, organomegaly, endocrinopathy, monoclonal gammopathy, and skin changes
PTH: Parathyroid hormone
SFTS: Severe fever with thrombocytopenia syndrome
SLE: Systemic lupus erythematosus
TAFRO: Thrombocytopenia, anasarca, fever, reticulin fibrosis, renal dysfunction, and organomegaly
TTP: Thrombotic thrombocytopenic purpura

## Declarations

### Human and animal participants

This article does not contain any studies with human participants or animals performed by any of the authors.
